# A Pilot Study of Home-Based Exercise and Personalized Nutrition Counseling Intervention in Endometrial Cancer Survivors

**DOI:** 10.3389/fonc.2021.669961

**Published:** 2021-06-11

**Authors:** Amanda R. Schwartz, David B. Bartlett, Johanna L. Johnson, Gloria Broadwater, Meghan Channell, Kimberly C. Nolte, Patricia A. Wilkes, Kim M. Huffman, Angeles Alvarez Secord

**Affiliations:** ^1^ Department of Obstetrics and Gynecology, Duke University Hospital, Durham, NC, United States; ^2^ Department of Medicine, Division of Medical Oncology, Duke University Hospital, Durham, NC, United States; ^3^ Duke Molecular Physiology Institute, Duke Center for Living, Duke University, Durham, NC, United States; ^4^ Department of Biostatistics and Bioinformatics, Duke Cancer Institute, Duke University Medical Center, Durham, NC, United States; ^5^ Department of Clinical Research, Duke Cancer Institute, Duke University Medical Center, Durham, NC, United States; ^6^ Division of Gynecologic Oncology, Department of Obstetrics and Gynecology, Duke Cancer Institute, Duke University Health System, Durham, NC, United States; ^7^ Department of Nutrition Services, Duke University Hospital, Durham, NC, United States; ^8^ Department of Medicine, Division of Rheumatology, Duke University Hospital, Durham, NC, United States

**Keywords:** endometrial cancer, obesity, cardiovascular disease, exercise intervention study, nutrition intervention program

## Abstract

**Introduction:**

To assess the feasibility of a home-based aerobic exercise and nutrition counseling intervention and effect on cardiorespiratory fitness, cardiovascular disease risk profile, and immune response in obese endometrial cancer survivors.

**Methods:**

A longitudinal pilot study assessed a 12-week home-based aerobic exercise and nutrition counseling intervention in obese endometrial cancer survivors. The primary outcome was feasibility defined as 80% adherence to weekly walking sessions calculated among individuals that completed the intervention. Secondary outcomes comprised pre- and post-intervention differences in cardiorespiratory fitness, cardiovascular risk factors, and T-cell function. Descriptive statistics summarized data. Wilcoxon sign tests identified differences between and pre and post-intervention variables.

**Results:**

Nineteen women with stage 1 endometrial cancer consented; 9 withdrew and one was a screen failure. Median adherence to weekly walking sessions was 83.3%. Body composition was significantly altered with a reduction in median fat mass from 52.5 kg to 46.9 kg (p=0.04), and BMI from 37.5 kg/m2 to 36.2 kg/m2 (p = 0.004). There was no significant difference in cardiorespiratory fitness or cardiovascular parameters. The percentage of CD4^+^ and CD8^+^ T-cells producing IFNγ towards MAGE-A4 significantly increased from and 5.9% to 7.2% (p=0.043) and 13.9% to 14.8% (p=0.046), respectively. There were 3 related adverse events: hip pain, back sprain, and abdominal pain.

**Discussion:**

Our home-based exercise and nutrition counseling program was feasible based on 80% adherence to walking sessions and favored altered body composition. However, the discontinuation rate was high and further research is needed to overcome barriers to implementation. Improvement in cardiovascular parameters will most likely require longer and more intensive programs.

## Introduction

Endometrial cancer is the sixth most common malignancy in women with over 380,000 new cases globally in 2018 (1). The majority of women are diagnosed with localized disease, and in the United States alone there are approximately 727,700 endometrial cancer survivors (2).

Endometrial cancer is an obesity-driven malignancy, with the alarming rates of obesity contributing to increases in incidence and mortality.

Obesity negatively impacts prognosis for endometrial cancer patients with a meta-analysis of women with endometrial cancer showing that a 10% increase in BMI increased odds of all-cause mortality by 9.2% (3). Calle et al. reported that morbidly obese (BMI > 40) endometrial cancer survivors had a 6.25 times increased risk of death compared to normal weight endometrial cancer survivors, with cardiovascular disease (CVD) being the leading cause of death (4). Felix and colleagues reported that women with endometrial cancer were 8.8 times more likely to die of CVD compared to women in the general population (5).

Therefore, we conducted a pilot study evaluating a 12-week home-based physical activity and nutrition counseling intervention to assess feasibility, safety, and effect on the cardiorespiratory fitness, CVD risk profile, and immune response endometrial cancer survivors.

## Materials and Methods

### Hypothesis and Study Objectives

We hypothesized that a 12-week home-based exercise and personalized nutrition counseling intervention will be both feasible and safe among endometrial cancer survivors. Our primary objective was to assess the feasibility of the intervention, defined as 80% adherence to prescribed weekly walking sessions among individuals that completed the intervention. Our secondary objectives were to evaluate the safety of the program as well as its effects on the cardiorespiratory fitness, cardiovascular risk factors and immune response.

### Patient Selection

Women with early stage localized endometrial cancer were recruited from the Duke Gynecologic Oncology clinic from May 2017 through July 2018. The inclusion criteria were as follows: age greater than 18 years; localized endometrial cancer (stage I and II); at least two months since hysterectomy and planned intervention date; Karnofsky performance status ≥ 70%; BMI 30.0 – 49.9; body weight < 300 pounds; no plan for adjuvant endometrial therapy; ability to exercise safely on a treadmill; reliable transportation; ability to provide informed consent; ability to speak and understand English; and ownership of a personal mobile device compatible with the study activity monitor. Individuals scheduled to undergo adjuvant endometrial therapy were excluded secondary to concern that further therapies may limit their ability to participate in scheduled exercise. Patients were excluded if they were already participating in more than 60 minutes of moderate intensity or 30 minutes of vigorous intensity exercise per week, or weight reduction dieting. This exercise criteria, which is well below American Heart Association recommendations for adult physical activity, was selected in order to identify patients most likely to benefit from the intervention. Patients with any absolute contraindications to exercise testing based on American Thoracic Society criteria were excluded.

### Study Design

The pilot study consisted of 12 weeks of home-based exercise and nutrition intervention with pre and post-intervention testing. IRB approval was obtained (Pro00074542).

Eligible patients underwent pre-intervention cardiorespiratory fitness and laboratory testing and body composition measurements including BOD POD assessment of body fat percentage. BOD POD uses air displacement plethysmography to provide a precise scale and volume measurement of body composition.

As an exploratory analysis, pre and post-intervention T-cell function was assessed. Tumor-associated antigen (TAA) specific T-cells were generated by co-culturing peripheral blood mononuclear cells (PBMCs) with peptide pulsed dendritic cells generated from the THP-1 (ATCC, VA, USA) monocytic cell line similar to previous methods (6).

Exercise recommendations included 10,000 steps per day and five weekly walking sessions for 25-30 minutes at a moderate intensity, defined as being able to carry on a brief conversation, but not able to sing or a 12 – 14 on the Borg Rating of Perceived Exertion scale. Daily steps were recorded by the FitBit (San Francisco, CA) activity monitor and walking sessions recorded in the exercise diary. Nutrition counseling was provided by a trained dietician (PAW) with recommended individualized daily caloric intake based on adjusted body weight to achieve weight loss. Participants were contacted every few weeks to provide support, assess progress and identify safety concerns. After the 12-weeks, post-intervention testing and a qualitative assessment were performed.

Any adverse events that occurred during the study duration were reviewed and signed off by the principal investigator and reported to the IRB in accordance with current policies.

### Data Analysis and Statistics

The primary outcome of this longitudinal pilot study was feasibility, defined by 80% median adherence to weekly walking sessions among women completing the intervention. Adherence to weekly walking sessions was defined as walking 25 – 30 minutes at a moderate intensity as reported in the participant’s exercise diary. Based on previous exercise trial experience a dropout rate of 25% was anticipated with a plan to enroll 15 subjects to achieve program completion for 10 women. Safety of the study was assessed by the type and prevalence of adverse events during the course of the pilot study.

Descriptive statistics were used to summarize the data. Wilcoxon sign tests were used to test for differences between pre and post-intervention variables. Statistical analysis was conducted using SAS v. 9.4 software.

## Results

### Patient Selection and Demographics

**Figure 1:**
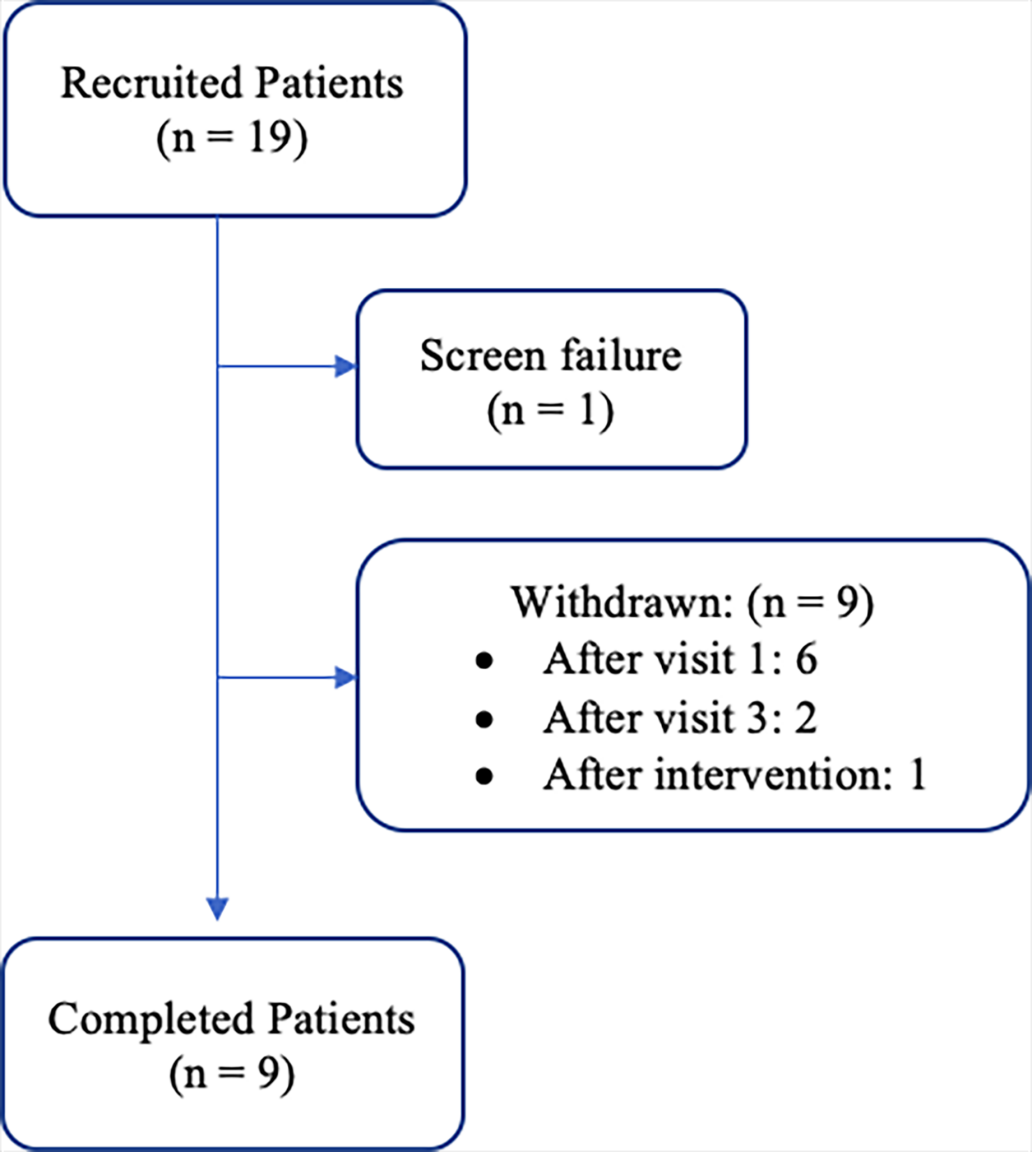
Patient cohort flow diagram: recruitment and retention of participants throughout the duration of the study period.

19 women meeting inclusion criteria were consented; one was a screen failure and 9 withdrew (**Figure 1**). Eight of the 9 women that withdrew from the study, discontinued during the pre-intervention period. Only one patient withdrew after initiation of the exercise and nutrition counseling intervention. Reasons for withdrawal included inability to commit to the study due to time requirements (n = 1), claustrophobia during BOD POD assessment (n = 1), and unknown (n = 7).

Nine patients enrolled and completed the study (**Table 1**).

**Table 1:** Patient Demographics.

Patient Characteristic	Patient cohort (n = 9)
Age in years, median (Range)	64.8 (57.8 – 71.1)
BMI in kg/m^2^, median (IQR)^1^	37.5 (35.2 – 39.5)
Race: N (%) Caucasian: African American Other:	6 (66.7) 3 (33.3) 0 (0.0)
Ethnicity: N (%) Not Hispanic or Latino Hispanic or Latino	9 (100.0) 0 (0.0)
Histology: N (%) Endometrioid	9 (100.0)
Grade: N (%) Grade 1: Grade 2:	8 (88.9) 1 (11.1)
FIGO Stage 2009: N (%) Stage IA	9 (100.0)
Performance Status: N (%) Karnofsky 90 Karnofsky 100	4 (44.4) 5 (55.6)

^1^IQR, interquartile range.

### Feasibility and Safety

The median adherence to prescribed weekly walking sessions for women completing the intervention was 83.3% (45.8% – 91.7%) and the mean adherence was 85.7% (41.6% - 100%) (**Table 2**). There were three study-related adverse events: abdominal pain (n = 1); back sprain (n = 1) and unilateral hip pain (n = 1). The abdominal pain and back sprain resolved on study. The participant with unilateral hip pain was lost to follow up.

**Table 2:** Activity and Nutrition Adherence.

Variable	Median (IQR)^1^
Adherence to weekly walking sessions (5 of 7 days walked)	83.3% (45.8% – 91.7%)
Days with a walking session (goal of 72%)	78.6% (56.0% – 90.5%)
Mean steps per day	9036 (8125 – 9806)
Total steps	759044 (607949 – 804371)
Mean daily calories burned	2000 (1700 – 2000)
Mean daily calories consumed	2700 (1800 – 3700)
	**Mean (range)**
Mean adherence to prescribed walking sessions (60 sessions)	85.7% (41.6% - 100%)

^1^IQR, interquartile range.

### Cardiorespiratory Fitness, Cardiovascular Disease Risk Factors

There were no significant differences from baseline to post-intervention markers of cardiorespiratory fitness or CVD. A significant decrease in total body mass was observed from visit 2 median body mass of 99.7 kg to visit 6 median body mass of 93.8 kg (p = 0.004; **Table 3**). Similarly, there was a significant decrease in total fat mass (p = 0.04) and BMI (p = 0.004).

**Table 3:** Pre and Post Intervention Markers of Cardiorespiratory Fitness, Cardiovascular Disease and Body Composition.

Variable, median (IQR)^1^	Pre-intervention	Post-intervention	Difference	P value
**Cardiorespiratory Fitness Markers**				
Peak aVO_2_^2^, in L/min	1.5 (1.4 – 1.7)	1.6 (1.4 – 1.7)	0.0 (-0.1 – 0.0)	0.73
Peak rVO_2_^3^, in mL/kg/min	16.3 (14.6 – 17.8)	16.7 (15.7 – 19.8)	-0.9 (-2.5 – 0.1)	0.45
Peak respiratory exchange ratio	1.1 (1.1 – 1.2)	1.1 (1.0 – 1.2)	0.0 (0.0 – 0.1)	1.00
Respiratory peak exchange	18.0 (17.0 – 19.0)	19.0 (17.0 – 19.0)	0.0 (-2.0 – 0.0)	0.13
Resting systolic BP, in mm Hg	122 (122 – 126)	120 (114 – 118)	4.0 (0.0 – 6.0)	0.29
Resting diastolic BP, in mm Hg	78 (72 – 80)	78 (74 – 86)	0.0 (-4.0 – 2.0)	1.00
Time to exhaustion, in seconds	375 (327 – 471)	418 (372 – 454)	-43.0 (-79.0 – -12.0)	0.07
**Cardiovascular Disease Risk Factors**				
C-reactive protein, in mg/L	4.2 (3.0 – 6.1)	4.4 (2.6 – 6.3)	0.4 (-0.2 – 1.1)	0.73
Cholesterol, in mg/dL	195 (171 – 216)	196 (153 – 236)	-1.0 (-14.0 – 9.0)	1.00
HDL^4^, in mg/dL	50 (38 – 67)	61 (36 – 65)	-2.0 (-3.0 – 2.0)	0.73
LDL^5^, in mg/dL	117 (71 – 132)	116 (102 – 151)	-4.0 (-19.0 – 6.0)	1.00
TG^6^, in mg/dL	112 (90 – 124)	106 (68 – 114)	13.0 (5.0 – 25.0)	0.18
**Body Measurements and Composition**				
Body Mass, in kg	99.7 (97.3 – 104.2)	93.8 (91.8 – 99.4)	4.0 (3.5 – 5.5)	**0.004**
Body Mass Index, in kg/m^2^	37.5 (35.9 – 41.2)	36.2 (34.1 – 39.0)	2.3 (1.4 – 2.9)	**0.004**
Fat mass, in kg	52.5 (45.0 – 54.7)	46.9 (43.4 – 50.8)	2.1 (1.4 – 5.8)	**0.04**
Hip circumference, in cm	123.4 (111.6 – 127.3)	122.8 (113.3 – 127.3)	2.5 (1.1 – 3.3)	0.18
Lung volume, in liters	3.3 (3.1 – 3.3)	3.3 (3.1 – 3.3)	0.0 (0.0 – 0.0)	–
Percentage body fat	51.6 (50.6 – 54.0)	51.1 (48.3 – 55.0)	0.0 (-1.0 – 1.8)	1.00
Percentage lean mass	48.4 (45.5 – 49.4)	51.0 (48.6 – 53.0)	-0.9 (-2.6 – 0.8)	0.73
Waist circumference, in cm	118.6 (114.8 – 122.3)	112.9 (111.3 – 117.5)	3.5 (1.1 – 5.7)	0.18
**Immune Function**				
CD4+ T-cells (% of Lymphocytes)	60 (57 – 62)	61.5 (57 – 62.5)	1.0 (-0.5 – 1.3)	0.257
CD4+/IFNγ+ (%)				
MAGE-A3	6.9 (4.9 – 7.4)	7.2 (5.8 – 9.1)	1.0 (0.2 – 2.0)	0.080
MAGE-A4	6.0 (4.5 – 7.2)	7.2 (5.3 – 9.2)	0.8 (0.9 – 2.2)	**0.043**
CD8+ T-cells (% of Lymphocytes)	34.9 (32.5 – 36.7)	36.3 (32.3 – 37)	0.3 (-1.0 – 1.6)	0.500
CD8+/IFNγ+ (%)				
MAGE-A3	15.2 (13.9 – 15.8)	15.2 (14.9 – 17.7)	1.3 (0.2 – 2.1)	0.080
MAGE-A4	13.4 (13.1 – 15.1)	15.3 (13.6 – 15.8)	0.9 (0.3 – 1.5)	**0.043**

^1^IQR, interquartile range.

^2^aVO2, absolute peak oxygen consumption.

^3^rVO2, relative peak oxygen consumption.

^4^HDL, high density lipoprotein.

^5^LDL, low density lipoprotein.

^6^TG, triglyceride.

In bold: Statistically significant (p < 0.05).

### Immune Function

Of the nine participants, blood samples for immune assessment were available for five women, and samples were not available for four [processing issue (n = 2); limited venous access (n = 2)]. Following a six-hour stimulation, the percentage of CD4^+^ and CD8^+^ T-cells producing IFNγ towards MAGE-A4 significantly increased from and 5.9% to 7.2% (p=0.043) and 13.9% to 14.8% (p=0.043), respectively (**Table 3**).

### Qualitative Assessment

All nine women completing the study reported a positive experience on the telephone survey. Emerging themes in qualitative assessment of the study included increases in self-confidence, motivation, perception of energy levels and exercise tolerance (**Table 4**).

**Table 4:** Free responses to qualitative prompts and corresponding themes.

Emerging theme	Patient quotes
Increased self-confidence	“I lost a lot of weight and people started to notice. I feel wonderful and I’m very happy about my progress” “I started in a discouraged place because of my past weight however this study helped me”
Increased energy levels and exercise tolerance	“I never thought I would be able to walk 10,000 steps and I was frequently short of breath during normal walking activities, but now I am able to do these activities with ease” “I can walk longer distances and not get tired as I once did before” “I feel more energized because of this study”
Increased motivation and desire to continue beyond the 12-week intervention	“Motivated me to increase my steps and reach my weekly goals. I really enjoyed this program and will continue with it” “Had me thinking I want to be around more for my granddaughter and want to continue eating health and exercising. I plan to continue using my Fitbit” “I’m going to continue using the tools I learned going forward” “The results from the first BODPOD test challenged me to improve. I’m going to continue walking and using my Fitbit”
Desire for more individualized nutrition counseling	“I wanted more contact with the dietician throughout the study” “The Fitbit held me accountable with exercise but I didn’t feel there was much accountability regarding the nutritional aspect” “I suggest more 1-on-1 conversations to offer tips on nutrition and how to lose weight” “I desired a meal plan more tailored to my calorie goal” “More individualized … the dietician should find out the person’s actual diet and strategize on meal plans that would work for that person”

## Discussion

Our findings demonstrated that a home-based exercise and nutrition counseling program is feasible based on 80% adherence to walking sessions and favored altered body composition in those initiating the study exercise and nutrition intervention. The walking sessions were safe and there were no serious or life-threatening events. There was a high rate of drop-out during the study pre-intervention phase of the study which included the baseline cardiorespiratory testing and BODPOD measurements. Given this high rate of discontinuation prior to the exercise and nutrition intervention, and lack of information regarding reason for withdrawal, further research is needed to overcome barriers to implementation.

No serious or life-threatening adverse events occurred during this study. The musculoskeletal injuries reported were likely secondary to increased activity and were similar to adverse events reported in previous weight-loss interventions (7–9). In addition, our findings revealed a significant decrease in total body mass, fat mass, and BMI.

There were no significant improvements in markers of cardiorespiratory fitness or other CVD risk factors observed. The presence of baseline EKG abnormalities in seven of nine patients was alarming and further highlights the need for intervention to improve the cardiovascular health of these women.

Our novel exploratory analyses of T-cell function show a significant improvement in IFNγ production from CD^4+^ and CD8^+^ T-cells towards the tumor associated antigen MAGE-A4, and a non-significant trend for MAGE-A3 suggesting that T-cells post-intervention are more capable of recognizing tumor antigens. In these women there was a significant increase in relative VO_2peak_ (p=0.043) suggesting a relationship with cardiorespiratory fitness and immune function. In healthy individuals, a single bout of exercise generated an enhanced T-cell response to tumor antigens, suggesting a beneficial role for exercise and tumor control by T-cells (6). Whether improved T-cell response will result in reduced risk for endometrial cancer recurrence remains unclear. Though the mechanisms remain unknown, our findings suggest physical activity and diet may enhance cytotoxic T-cell responses to cancer antigens. A previous investigation of endometrial morphology and molecular pathways following bariatric surgery-induced weight loss demonstrated reduction in biomarkers of insulin resistance (hemoglobin A1C, HOMA-IR) and inflammation (hsCRP, IL-6) demonstrating potential mechanisms by which weight loss may impact endometrial cancer risk (10). Another investigation of biomarkers in obese women following bariatric surgery similarly demonstrated reduction in inflammatory markers, including CRP, IL-1Ra and IL-6, following weight loss (11). The reduction in inflammatory markers following bariatric surgery suggest that weight loss contributes to a favorable immune environment that may restore the endometrium to a lower risk state. Our findings of an enhanced cytotoxic T-cell response following an exercise and nutrition counseling intervention further suggest a possible role of exercise and/or nutrition on immunologic response. Further investigation is needed to provide insight in potential regulatory mechanisms of exercise, nutrition, and/or weight loss on immune function.

Previous weight-loss interventions in other patient populations at increased risk of CVD have shown improvements in markers of cardiovascular fitness and CVD risk factors (12, 13). While improvement in body mass was seen in our pilot cohort, we believe that the lack of favorable changes on markers of cardiorespiratory fitness and lipid profile was secondary to the small sample size and short-term intervention.

Among endometrial cancer survivors, there have been limited high-quality exercise and nutrition counseling interventions. The SUCCEED trial, a six-month nutrition and exercise intervention among 70 obese endometrial cancer survivors, demonstrated an increase in physical activity and weight loss (7). Basen-Engquist and colleagues reported a home-based exercise intervention in endometrial cancer survivors which showed increased physical activity, improved predicted VO_2MAX_, and improved systolic blood pressure among the obese cohort (14). While these studies have shown promising results, a recent Cochrane analysis of interventions for weight loss in endometrial cancer survivors found no improvement in cancer-specific survival, overall survival, cardiovascular event frequency, or significant weight loss at six or twelve months (15).

The qualitative survey at the conclusion of the study indicated that the intervention was well received with all nine women reporting their experience to be beneficial and four specifically noting plans to continue to apply the lifestyle changes beyond the intervention period. Previous investigations of weight-loss interventions in endometrial cancer survivors have likewise shown improvements in self-efficacy and emotional well-being which correlate with weight loss (8, 12, 16, 17). The qualitative assessment was performed at the conclusion of the study and therefore qualitative data was limited to only those individuals that completed the intervention. Therefore, responses are more likely positive than from those who did not complete the intervention.

Key limitations of the study included small sample size and participant dropout. Improvements in cardiorespiratory fitness and markers of CVD will most likely require longer and more intensive intervention. Although feasibility criteria was met among individuals that initiated the exercise and nutrition intervention study phase, the generalizability of this pilot investigation is significantly limited by the small sample size. The 50% dropout rate is higher than in similar interventions aimed at weight loss in endometrial cancer survivors (19-22%) and raises concern for withdrawal bias (8, 9). This exceeded our estimated dropout rate of 25% and ultimately resulted in a lower number of pilot study participants than originally anticipated. Patients were screened only by initial interest with many women choosing not to enroll after the study requirements were described in further detail and other women enrolling in the study but later being unable or unwilling to complete the study tasks. Given that the majority of the women that withdrew from the study did so prior to initiation of the exercise and nutrition counseling intervention, more thorough counseling of the pre-intervention testing requirements may contribute to lower rates of discontinuation in future trials. The more intensive requirement for cardiopulmonary exercise testing as well as the pre and post-intervention testing requiring additional transportation may have contributed to the higher dropout rate. Despite a high drop-out rate, the results of the qualitative assessment indicate that the intervention was well-received among the women that completed the study. Unfortunately, we are unable to identify the patients that are more likely to successfully complete the intervention at this point in time. Further measures to mitigate patient discontinuation, such as more detailed discussion of study participation at time of enrollment, centralization of study visits to a single site or closer follow-up throughout the duration of the study would need to be considered prior to application of this pilot intervention on a broader scale.

In conclusion, our pilot study represents a feasible and safe method of home-base physical activity and nutrition counseling intervention for endometrial cancer survivors which led to significant improvements in weight and BMI. However, longer and more intensive studies will be needed to determine if exercise and nutrition weight loss interventions will reduce the risk of CVD and mortality in endometrial cancer survivors. Our results will inform future study designs and support funding to conduct further studies on cardiovascular biomarkers and cardiovascular fitness in endometrial cancer survivors.

## Data Availability Statement

The raw data supporting the conclusions of this article will be made available by the authors, without undue reservation.

## Ethics Statement

The studies involving human participants were reviewed and approved by Duke Institutional Review Board. The patients/participants provided their written informed consent to participate in this study.

## Author Contributions

AAS and KH played an active role in the design of the pilot study. The IRB was drafted by AAS. AAS, KH, and ARS participated in the obtaining of funding for the study. AAS, JJ, KN and MC participated in patient recruitment. PW created a personalized nutrition plan for each patient and completed individualized nutrition counseling. JJ and MC were integral to patient recruitment, calling patients throughout the study period and recording patient data. Exploratory analysis of T-cell function was performed by DB. Statistical analysis was performed by GB and DB. The research manuscript was drafted by ARS and AAS and edited by all listed authors. All authors contributed to the article and approved the submitted version.

## Funding

Charles B Hammond Research Fund (awarded 12/2017), Duke University School of Medicine, Durham, NC.

## Conflict of Interest

AAS reports grants from AbbVie, Amgen, Astex Pharmaceuticals Inc., Astra Zeneca, Clovis, Astellas Pharma Inc., Boehringer Ingelheim, Bristol Myers Squibb, Clovis, Eisai, Endocyte, Exelixis, Incyte, Merck, PharmaMar, Immutep Ltd, Roche/Genentech, Seattle Genetics, Inc, TapImmune, and Tesaro outside of submitted work. She reports honoraria from advisory boards with Alexion, Aravive, Astex Pharmaceuticals Inc., Astra Zeneca, Cordgenics, Clovis, Janssen/Johnson & Johnson, Merck, Mersana, Myriad, Oncoquest, Roche/Genentech, and Tesaro outside the submitted work.

The remaining authors declare that the research was conducted in the absence of any commercial or financial relationships that could be construed as a potential conflict of interest.
